# New insights into the potential cardioprotective effects of telmisartan and nanoformulated extract of *Spirulina platensis* via regulation of oxidative stress, apoptosis, and autophagy in an experimental model

**DOI:** 10.3389/fphar.2024.1380057

**Published:** 2024-05-09

**Authors:** May Almukainzi, Thanaa A. El-Masry, Hanaa A. Ibrahim, Hebatallah M. Saad, Enas I. El Zahaby, Asmaa Saleh, Maysa M. F. El-Nagar

**Affiliations:** ^1^ Department of Pharmaceutical Sciences, College of Pharmacy, Princess Nourah bint Abdulrahman University, Riyadh, Saudi Arabia; ^2^ Department of Pharmacology and Toxicology, Faculty of Pharmacy, Tanta University, Tanta, Egypt; ^3^ Department of Pathology, Faculty of Veterinary Medicine, Matrouh University, Matrouh, Egypt; ^4^ Department of Pharmaceutics, Faculty of Pharmacy, Delta University for Science and Technology, Gamasa, Egypt

**Keywords:** cyclophosphamide, *Spirulina platensis*, cardiotoxicity, PON-1, autophagy, nanoformulation, telmisartan

## Abstract

**Background:**

Cardiotoxicity is one of the limiting side effects of the commonly used anticancer agent cyclophosphamide (Cyclo).

**Materials and methods:**

The possible protective effects of telmisartan and nanoformulated *Spirulina platensis* (Sp) methanolic extract against Cyclo-induced cardiotoxicity were examined in this study. Experimental groups of rats were randomly divided into nine groups as control vehicle, control polymer, telmisartan (TEL, 10 mg/kg), free Sp extract (300 mg/kg), nano Sp extract (100 mg/kg), Cyclo (200 mg/kg), TEL + Cyclo, free Sp + Cyclo, and nano Sp + Cyclo. The groups with Cyclo combinations were treated in the same manner as their corresponding ones without Cyclo, with a single dose of Cyclo on day 18.

**Results:**

The results indicate that Cyclo causes significant cardiotoxicity, manifesting in the form of notable increases of 155.49%, 105.74%, 451.76%, and 826.07% in the serum levels of glutamic oxaloacetic transaminase (SGOT), lactate dehydrogenase (LDH), creatine kinase MB (CK-MB), and cardiac troponin I (cTnI) enzyme activities, respectively, as compared to the control. In addition, the cardiac glutathione (GSH) content and activity of glutathione peroxidase-1 (GPX-1) enzyme decreased by 65.94% and 73.85%, respectively. Treatment with nano Sp extract showed the most prominent restorations of the altered biochemical, histopathological, and immunohistochemical features as compared with those by TEL and free Sp; moreover, reductions of 30.64% and 43.02% in the p-AKT content as well as 60.43% and 75.30% of the endothelial nitric oxide synthase (eNOS) immunoreactivity were detected in the TEL and free Sp treatment groups, respectively. Interestingly, nano Sp boosted the autophagy signal via activation of beclin-1 (36.42% and 153.4%), activation of LC3II (69.13% and 195%), downregulation of p62 expressions (39.68% and 62.45%), and increased gene expressions of paraoxonase-1 (PON-1) (90.3% and 225.9%) compared to the TEL and free Sp treatment groups, respectively.

**Conclusion:**

The findings suggest the protective efficiency of telmisartan and nano Sp extract against cardiotoxicity via activations of the antioxidant, antiapoptotic, and autophagy signaling pathways.

## Graphical Abstract

**Figure:**
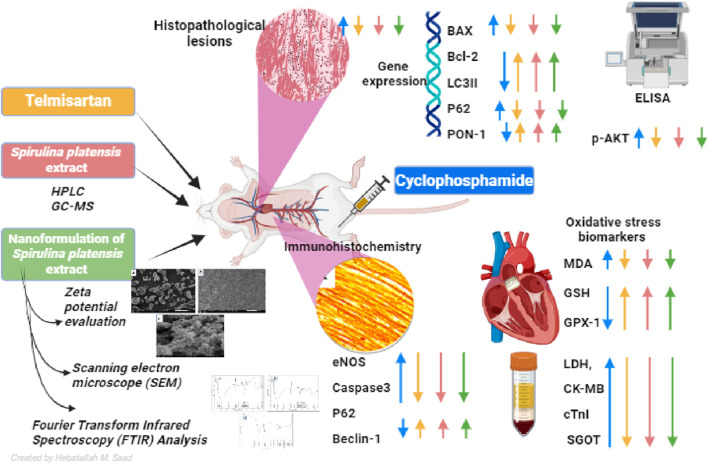

## 1 Introduction

Several pharmacological treatments have been developed against tumors as a result of their increasing incidence. However, some of these medications cause cardiotoxicity, which poses serious problems for medical personnel, as DNA is alkylated by the nitrogen mustard medication cyclophosphamide (Cyclo). At dosages exceeding 120–150 mg/kg, the incidence of cardiotoxicity is 8%–20% in the elderly and 5% in children. Heart failure (HF) is generally caused by Cyclo at rates ranging from less than 5% to up to 10%–29% (Auner et al., 2002; Zver et al., 2008).

Cyclo is transformed into an active form *via* DNA and RNA crosslinking, which prevents protein synthesis and is not phase-specific to the cell cycle (Mills et al., 2019; Almeida de Oliveira et al., 2022); when coupled with glutathione, it produces reactive oxygen species (ROS) that hinder the efficiency of the antioxidant mechanism (Tsai-Turton et al., 2007). In addition, malondialdehyde (MDA), a lipid peroxidation marker, increases following Cyclo therapy (Tsai-Turton et al., 2007). However, its most hazardous metabolite is acrolein (Kurauchi et al., 2017), which is a highly reactive unsaturated aldehyde that causes the cardiac tissue to become particularly sensitive (Henning et al., 2017). In addition to having a significant inflammatory impact on cardiomyocytes, Cyclo and/or its metabolites interact with proteases to produce oxygen free radicals. Nowadays, antioxidants are being researched as possible remedies for the adverse effects of Cyclo and as a means of reducing ROS (Ayza et al., 2020) because antioxidant compounds can minimize the biochemical and physiological issues associated with Cyclo (Afkhami-Ardakani et al., 2017).

*Spirulina platensis* (or *Arthrospira platensis*, Sp) is a filamentous cyanobacterium comprising minerals (particularly iron), vitamins, essential fatty acids, proteins, carbohydrates, and pigments. Sulfated polysaccharides, γ-linolenic acid, proteins, and phycocyanin are the main bioactive constituents that appear to be important in promoting better functions of the human body (Wang et al., 2005). Medical experts are interested in spirulina because of its unique qualities, which include antiviral, anticancer, anti-inflammatory, antiapoptotic, antioxidant, and immunomodulatory effects (Parham et al., 2020; Fais et al., 2022; Jongrungraungchok et al., 2023).

Chitosan has also been proven to be non-toxic, biodegradable, and biocompatible; it is insoluble in water but soluble in acidic solvents, such as diluted hydrochloric, formic, and acetic acids. It contains a primary amine group to which the majority of biological effects are attributed (Fatullayeva et al., 2022). Chitosan is a biocompatible, biodegradable, and naturally occurring biopolymer that is a deacetylated version of chitin, containing glucosamine and N-acetyl glucosamine units. This biopolymer exhibits various biological activities, including mucoadhesion, antimicrobial, immunostimulant, and wound healing capabilities as well as anticancer effects. Chitosan shell has been examined as a viable nano/submicron carrier system for controlled/long-term drug release because it is small enough to overcome the biological barrier. Chitosan’s versatility allows it to encapsulate both lipophilic and hydrophilic molecules, making it widely used in biomedical and pharmaceutical applications (Pulicharla et al., 2016).

The biological effects are correlated with a higher degree of chitosan deacetylation, and low-molecular-weight chitosan has been shown to have greater biological effects (Kim, 2018). The abundance of amino and hydroxyl groups in chitosan is responsible for its internal properties, such as mucoadhesion, controlled drug delivery, and permeability promotion (Harugade et al., 2023). Ionic gelation is simple and flexible method for the preparation of nanoparticles with high encapsulation efficiency for several medical and biological applications. Interferon and doxorubicin are examples of drugs that have been successfully encapsulated using this technique (Bocchetta, 2020). Several factors affect the properties of the synthesized nanoparticles, such as molecular weight/concentration of chitosan, chitosan to sodium tri poly phosphate (NaTPP) ratio, pH, centrifugation, stirring speed, and encapsulated drug concentration (Wang et al., 2016).

Chitosan nanoparticles can be produced by ionic gelation using chitosan and NaTPP; TPP is frequently used as a crosslinking agent in the preparation of chitosan nanoparticles as it is non-toxic, is multivalent, and produces gels through ionic interactions. The amount of crosslinker utilized in the manufacturing of the nanoparticles can result in smaller particles (Wang et al., 2016). The sizes of the nanoparticles have essential influences on their intracellular absorption, intracellular trafficking, and behaviors in biological fluids. The particle size also affects the *in vivo* biodistribution, stability, drug loading and release, and toxicity of the nanoparticles (Hoang et al., 2022).

Angiotensin receptor blockers (ARBs) are known to be safe and efficient antihypertensive medications that prevent the negative effects of the level of angiotensin II, a hormone that contributes to fibrosis and heart dysfunction, at the type 1 receptor (AT1) (Hasegawa et al., 2011). Telmisartan is one of the ARBs that has positive effects on hypertension-related cardiovascular and renal end-organ damage; it also helps maintain proper functioning of the mitochondria (Moore and Tabas, 2011). It has been shown to decrease interleukin (IL)-1β and tumor necrosis factor-α (TNF-α) as well as reverse the expressions of adhesion molecules and macrophage buildup (Jang et al., 2020).

Autophagy is a process by which damaged proteins and organelles are cleared from the heart cells, preventing their toxic buildup and promoting cellular health. It can counteract stress factors like inflammation and ROS that contribute to organ damage. In some cases, boosting autophagy has shown promise in protecting against toxicity caused by certain drugs or treatments (Gu et al., 2016). In the present work, the potential protective properties of telmisartan and chitosan nanoformulation of *Spirulina platensis* methanolic extract were evaluated against Cyclo-induced cardiotoxicity. Furthermore, their mechanistic actions were clarified in an experimental model of cardiotoxicity.

## 2 Materials and methods

### 2.1 Materials

Extra-pure low-molecular-weight chitosan 10–150 m. Pas with 90% DA (molecular weight less than 150 kD) was obtained from Sisco Research Laboratory (India) and NaTPP was obtained from Lanxess Company (India); high-analytical-grade glycol, polysorbate 80, glacial acetic acid, and NaOH were also procured similarly. Ion-free water (Stakpure, Waters, United States of America), analytical-grade methanol (Merck, Darmstadt, Germany), and cyclophosphamide (Endoxan (1 g), Astra Medica Co., Egypt) were also purchased. Telmisartan was provided by Sigma-Aldrich (United States, and all other agents obtained were of analytical grade and good quality.

### 2.2 Cultivation of *Spirulina platensis* (Sp)

A modified version of Zarrouk’s medium was used to cultivate Sp (Aiba and Ogawa, 1977). A temperature of 27°C ± 3°C and a light intensity of 45 µmol photons/m^2^s were maintained in the axenic culture flasks. The culture was supplied with a mixture of 3% CO_2_ and 97% dry-filtered air to accelerate growth.

Using a Sigma 2-16 KL Centrifuge, the culture was produced by centrifugation for 15 min at 4,000 rpm. Following two washings, the cultures underwent a 12-h freeze-drying process (Rogers and Burns, 1994). They were then stored at −20°C until use.

### 2.3 Preparation of Sp methanolic extract

Sp lyophilized powder (10 g) was added to 100 mL of methanol and shaken at 25°C for 72 h (VS-8480). A rotary vacuum evaporator was next used to concentrate the collected supernatant after extraction and freeze-drying, resulting in the final Sp freeze-dried methanolic extract powder. Thereafter, the sample was maintained at 4°C and stored away from light until it could be used as per a modified procedure of Al-Homaidan et al. (2015).

### 2.4 Sp methanolic extract gas chromatography–mass spectrometry (GC-MS) analysis

The Trace GC-ISQ mass spectrometer (Thermo Scientific, USA) was utilized as the GC-MS apparatus to analyze the chemical composition of the Sp methanolic extract. The temperature was varied from 50°C to 280°C at the rate of 10°C/min. The injector, interface, and source temperatures were 200°C, 220°C, and 220°C, respectively. El-Kareem et al. (2016) showed that helium could be employed as a carrier gas at a flow rate of 1 mL/min. The components were estimated and identified by comparing their retention lengths and mass spectra with information from the WILEY mass spectral database (Wiley Registry of Mass Spectral Data, ninth edition, Version 1.02) and NIST 05 (NIST/EPA/NIH mass spectral library version 2.0d).

### 2.5 Preparation of Sp nanoparticles (nano Sp) by ionic gelation

Crosslinked nanospheres were formulated using NaTPP and chitosan through a slight modification of a previously published method (Hoang et al., 2022). Chitosan (1 g) was dissolved in 1% acetic acid with the aid of a magnetic stirrer for 30 min (200 rpm) to prepare a chitosan solution (2% w/v), and the solution temperature was adjusted to 50°C. *Spirulina* powder (1 g) was next mixed with 1 mL of glycerol to produce a smooth paste, and 1% polysorbate (10 mL) solution was added stepwise with the aid of a prop sonicator (Sonic Vibra Cell, United States) for 4 min (cycles of 12 s pulse and 6 s break) at 65% power (130 W) to obtain a homogenous dispersion. The container was then immersed in an ice bath.

The free Sp dispersion was added stepwise to the chitosan solution and agitated for 60 min at room temperature (50 rpm). The free Sp/chitosan mixture was then transferred to a special amber glass container (prefreezed for 4 h at −80°C) and immersed in an ice bath. The mixture was sonicated for 6 min, and its pH was adjusted to 5 with the help of 2% NaOH. Using a 5-mL syringe, NaTPP (2.5% w/v) was dropped on the free Sp/chitosan mixture and continuously agitated (50 rpm); after the addition of NaTPP (4 mL), the mixture was further agitated for 30 min (50 rpm at 4°C). An ultracentrifuge (Centurion Scientific, United Kingdom) was used to separate the formulated nanoparticles (nanospheres) for 10 min at a speed of 10,000 rpm (−4°C). The precipitate mass was collected and rinsed twice with deionized water. The washed precipitate was lastly dried with a freeze dryer (Christ Benchtop Freeze dryer, Germany).

### 2.6 Sp nanoparticle characterization

#### 2.6.1 Yield percentage and entrapment efficiency (EE)

The efficiency of the formulation process was evaluated through its calculated yield percentage. The weights of all the ingredients (free Sp, chitosan, and NaTPP) were determined using a precalibrated six-digit analytical balance (Sartorious, United States). After lyophilization, the remaining powder was weighed again, and the percentage yield was estimated using the formula below.

% yield = [remaining powder (Sp nanoparticles)/ total weight of all ingredients] ×100

The drug EE was calculated by an indirect technique. After centrifuging the Sp nanoparticle formulation, the unentrapped percentage of the drug in the supernatant was evaluated using a calibration curve at a maximum absorbance wavelength of 235 nm corresponding to the lipid content of the alcoholic spirulina extract (Angioni et al., 2002). To reduce handling errors, the complete analysis was performed three times. The percentage of medication successfully encapsulated in the system is referred to as EE (Rehman et al., 2017).

The following formula can be used to determine EE:

$$\%\text{EE}=\left(\left(\text{total}\;\text{drug}\;\text{conc}.-\text{supernatant}\;\text{drug}\;\text{conc}.\right)/\left(\text{total}\;\text{drug}\;\text{conc}.\right)\times 100\%.\right)$$

#### 2.6.2 Average particle size, zeta potential, and polydispersity index

The particle size is the most important factor affecting the efficiency of the Sp nanoparticles, while the zeta potential indicates the extent of colloidal stability (Tahir et al., 2017).

The PDI describes the distribution of nanoparticle sizes; a nanoparticle system with a PDI <0.1 is considered to be very monodisperse, while PDIs >0.4 and in the range of 0.1–0.4 indicate highly polydisperse and moderately disperse distributions, respectively (Kane et al., 2016).

Experiments were performed in triplicate in deionized water at ambient temperature; the Zeta Sizer Nano (Malvern Panalytical Ltd., United Kingdom) was employed to determine the particle size and zeta potential.

#### 2.6.3 Scanning electron microscopy (SEM)

The surface characteristics and structure of the free Sp and nano Sp were evaluated using SEM. The lyophilized powder was dissolved in ethanol, sonicated, and one drop was disseminated over a glass slide. After complete drying, the power was transferred on top of a metal stub (cupper) on a silicon electroconductive chip. The sample was covered with a gold coat for 1 min so as to be ready for inspection at various magnifications using a 10 kV electron acceleration voltage field-emission scanning electron microscope (JEOL, JSM-6510LV, Japan).

#### 2.6.4 Fourier-transform infrared (FTIR) spectroscopy

The FTIR spectroscopy study was performed to test the interactions or compatibility of the formulation’s components to demonstrate the system stability (Jain et al., 2014). The powdered sample was mixed with dry potassium bromide (KBr) pellets and subjected to a pressure of approximately 5 × 106 Pa in an evacuated die. This resulted in a clear translucent disc of diameter 13 mm and thickness 1 mm. The IR spectra in the −4,000 to 400 cm^−1^ range were obtained at room temperature. A Perkin-Elmer Fourier-transform spectrometer fitted with an air-cooled deuterated triglycine sulfate detector was used (Theivandran et al., 2015). The free Sp and nano Sp powders were then analyzed using FTIR spectroscopy with a Bruker instrument (United States).

### 2.7 *In vivo* experiment

#### 2.7.1 Animals and ethical declaration

Male Wistar rats aged 6–8 weeks (180–200 g) were purchased from the Egyptian National Research Center. The rats were allowed unrestricted access to food and water at a constant room temperature. The procedures for the animal experiments adhered to the guidelines of the research ethics committee of the Faculty of Pharmacy, Tanta University, Egypt, as well as the standards of the Council for International Organizations of Medical Sciences (CIOMS) (Approval code: TP/RE/1/24p-01).

#### 2.7.2 Animal experiment protocols

After 14 days of acclimatization, the animals were divided into nine groups (Gp) with six rats each as follows: Gp I (VC): intragastric administration of saline for 21 days; Gp II (PC): intragastric administration of chitosan polymer for 21 days; Gp III (TEL): intragastric administration of 10 mg/kg of telmisartan (TEL) for 21 days (Ali et al., 2022); Gp IV (free Sp): intragastric administration of 300 mg/kg of free Sp extract for 21 days (Khalil et al., 2020); Gp V (nano Sp): intragastric administration of 100 mg/kg of nano Sp extract for 21 days (Hardiningtyas et al., 2022); Gp VI (Cyclo): one intraperitoneal injection of 200 mg/kg Cyclo on day 18 of the experiment (Bhatt et al., 2017); Gp VII (TEL + Cyclo): intragastric administration of 10 mg/kg of TEL for 21 days plus one intraperitoneal injection of Cyclo (200 mg/kg) on day 18; Gp VIII (free Sp + Cyclo): intragastric administration of 300 mg/kg of free Sp extract for 21 days plus one intraperitoneal injection of Cyclo (200 mg/kg) on day 18; Gp IX (nano Sp + Cyclo): intragastric administration of 100 mg/kg of nano Sp extract for 21 days plus one intraperitoneal injection of Cyclo (200 mg/kg) on day 18.

#### 2.7.3 Collection of blood and heart tissue

Seventy-two hours after administration of Cyclo, the rats were anesthetized using 5% isoflurane in an induction chamber (Tsukamoto et al., 2016). The isoflurane anesthesia was administered through a rodent inhalant equipment (SomnoSuite Small Animal Anesthesia System, Kent Scientific Corporation, Connecticut, USA), and its flow rate was calculated according to the following formula: Flow rate (mL/min) = 0.65 × body weight (g). Blood samples were collected, and the hematology samples were centrifuged at 4,000 rpm for 15 min at 4°C via the Sigma 2-16 KL centrifuge for estimation of cardiac enzymes. The animals’ hearts were excised, and the tissues were fixed using 10% buffered formalin solution for histopathological and immunohistochemical analyses. The remaining tissue samples were stored at −80°C for biochemical investigations.

#### 2.7.4 Cardiac enzyme measurements

Serum samples were analyzed for serum glutamic oxaloacetic transaminase (SGOT), lactate dehydrogenase (LDH), and creatine kinase MB (CK-MB) using kits and kinetic methods as per manufacturer instructions. The kits were supplied by SPINREACT, Spain (catalog nos. MDBEIS46-I, BEIS16-E, and BEIS04-I). In addition, the serum level of cardiac troponin I (cTnI) enzyme was quantitatively evaluated using an ELISA kit purchased from Abcam Inc., United States (catalog no. ab246529).

#### 2.7.5 Antioxidant activities and lipid peroxidation measurements in cardiac tissue

Lipid peroxidation (MDA), reduced glutathione (GSH) content, and glutathione peroxidase (GPX-1) enzyme activity were assessed using various commercial ELISA kits provided by MyBioSource.co (CUSABIO Co., catalog nos. MBS268427, CSB-E12144r, and MBS9425463, respectively). All experimental procedures were performed according to manufacturer instructions.

#### 2.7.6 Determination of p-AKT

The p-AKT content in cardiac tissues was measured using the MyBioSource.co ELISA kit (catalog no. MBS7254603) as per manufacturer instructions.

#### 2.7.7 Gene expressions of Bax, Bcl2, PON-1, LC3II, and p62 via qRT-PCR

The gene expressions of Bax, Bcl2, PON-1, LC3II, and p62 genes were measured as per manufacturer protocols, and Table 1 shows the primer sequences that were employed. The ΔCt values were computed using the Rotor-Gene Q Series 2.0.3 (Build 2) software package. The cycle numbers are indicated by the Ct values once the fluorescence curves have reached the baseline values. By normalizing the control group and β-actin level, the relative mRNA expressions of the target genes were computed for fold changes using the 2^−ΔΔCt^ technique (Yuan et al., 2006).

**TABLE 1:** Primers sequence.

Gene	Forward 5′-3′	Reverse 5′-3′	Reference
*PON-1*	TGA​GAG​CTT​CTA​TGC​CAC​AAA​TG	CCA​TGA​CAG​GCC​CAA​GTA​CA	Hafez et al. (2014)
*Bax*	CAC​CAG​CTC​TGA​ACA​GAT​CAT​GA	TCA​GCC​CAT​CTT​CTT​CCA​GAT​GGT	Kinouchi (2003)
*Bcl-2*	CAC​CCC​TGG​CAT​CTT​CTC​CTT	AGC​GTC​TTC​AGA​GAC​AGC​CAG	Kinouchi (2003)
*LC3II*	CAT​GCC​GTC​CGA​GAA​GAC​CT	GAT​GAG​CCG​GAC​ATC​TTC​CAC​T	Zhang et al. (2015)
*P62*	GCC​CTG​TAC​CCA​CAT​CTC​C	CCA​TGG​ACA​GCA​TCT​GAG​AG	Zhang et al. (2015)
*β-actin*	TCC​TCC​TGA​GCG​CAA​GTA​CTC​T	GCT​CAG​TAA​CAG​TCC​GCC​TAG​AA	Banni et al. (2010)

#### 2.7.8 Histopathological examination and semiquantitative scoring

Heart tissue specimens from each experimental group were collected and preserved in a 10% solution of neutral buffered formalin. Subsequently, they were dehydrated using a high-concentration ethanol solution, followed by clearing with xylene. Finally, the samples were embedded in paraffin wax in accordance with routine processing protocols (Suvarna and Layton, 2018). Serial sections of 4 µm were sliced from the paraffin blocks and subsequently stained with hematoxylin and eosin (H&E). The degree and severity of the pathological damage were evaluated in a semiquantitative manner; this assessment was based on a scale where a score – was considered normal, ± was considered borderline, + was considered mild, ++ was considered moderate, and +++ was considered severe (El-Agamy et al., 2016).

#### 2.7.9 Immunohistochemical protein assay

Paraffin blocks from the heart tissues were dissected into thin slices of thickness 4 µm each. These slices were then subjected to immunostaining using the streptavidin-biotin peroxidase technique. In brief, the sections were incubated in citrate buffer at 95°C for 40 min for antigen retrieval. To prevent endogenous peroxidase activity, an addition incubation period of 10 min was employed along with a peroxidase blocker. To block any nonspecific binding sites, a 10% normal goat serum was applied for 10 min. Subsequently, the sections were incubated overnight at 4°C in a humidified chamber and treated with the following antibodies: anti-eNOS, anti-caspase-3, anti-beclin1, and anti-p62 (Santa Cruz Biotechnology Inc., Dallas, TX, United States). Horseradish peroxidase reagent and a secondary biotinylated antibody were applied to the slides for 30 min at 37°C. After each step, the slides rinsed thrice with phosphate-buffered saline. Subsequently, the sections were treated with 3,3′-diaminobenzidine tetrahydrochloride reagent for 3 min. To intensify the staining of the nuclei, the sections were counterstained with Mayer’s hematoxylin and ultimately mounted with DPX. For the quantitative histomorphometric analyses, ten randomly chosen micrographs were obtained from each section for eNOS, caspase-3, beclin-1, and p62. The percentage areas of the immunopositive slides were calculated from ten randomly selected fields from each rat in every group. Thereafter, it was represented as a percentage of positive area per square millimeter using ImageJ software (v1.46r, NIH, Bethesda, MD, United States) (Vis et al., 2000).

### 2.8 Statistical evaluations

Multiple comparisons were performed among the groups using one-way analysis of variance (ANOVA), and the results were regarded to be statistically significant at *p* < 0.05; this was followed by Tukey’s multiple comparisons tests using GraphPad Prism (GraphPad Software Inc., United States).

## 3 Results

### 3.1 *In vitro* analyses

#### 3.1.1 Analysis of Sp methanolic extract by GC-MS

**FIGURE 1:**
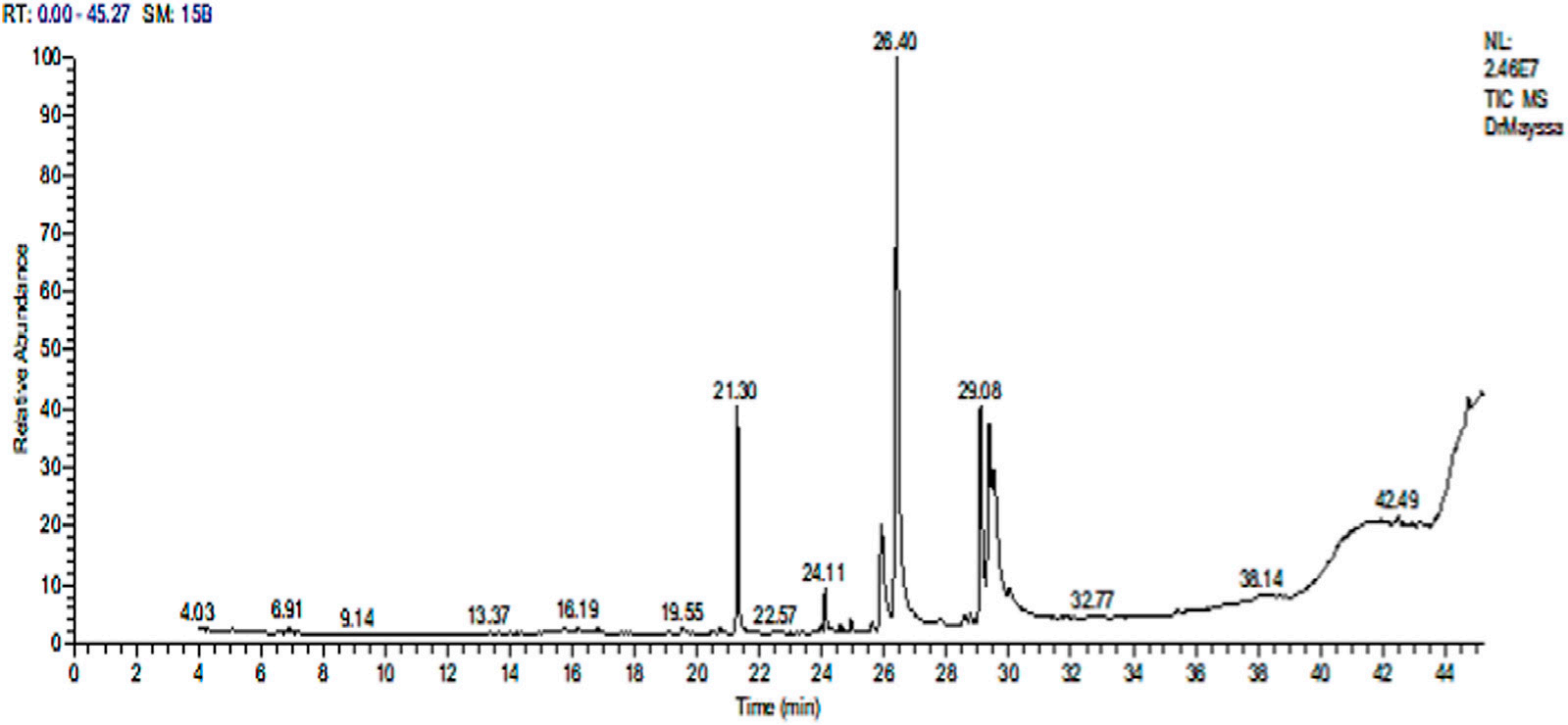
GC-MS chromatogram of the Sp methanolic extract.

Various compounds with wide ranges of biological activities were observed in the Sp methanolic extract, as shown in Figure 1. Table 2 lists the bioactive properties of these compounds, which primarily contained hexadecanoic acid (28.29%), 9,12-octadecenoic acid (Z) (22.29%), gamolenic acid (9.27%), palmitoleic acid (6.71%), heptadecane (6.44%), and 9-octadecenoic acid (Z) (4.48%).

**TABLE 2:** GC-MS analysis of the Sp methanolic extract*.

RT	Compound name	PA %	MF	Biological activity
21.30	Heptadecane	6.44	C_17_H_36_	Antioxidant, anti-inflammatory, and anticancer activities (Wu et al., 2005; Lin et al., 2010)
24.11	Phytol, acetate	1.32	C_22_H_42_O_2_	Antioxidant, anticancer, antimicrobial, and anti-inflammatory properties (Islam et al., 2018)
25.91	Palmitoleic acid	6.71	C_16_H_30_O_2_	Antioxidant, antibacterial, and anti-inflammatory activities (Agoramoorthy et al., 2007; Zadeh Hashem et al., 2016)
26.40	Hexadecanoic acid	28.29	C_16_H_32_O_2_	Antioxidant, antimicrobial, and hypocholesterolemic agent (Mazumder et al., 2020)
29.08	Gamolenic acid	9.27	C_18_H_30_O_2_	Anticancer and anti-inflammatory activities (Kapoor and Huang, 2006; Jóźwiak et al., 2020)
29.36	9,12-Octadecadienoic acid (Z,Z)	9.43	C_18_H_32_O_2_	Antioxidant and antimicrobial activities (Muzahid et al., 2023)
29.50	9,12-Octadecadienoic acid (Z,Z)	12.86	C_18_H_32_O_2_	As mentioned
30.01	9-Octadecenoic acid (Z)-	1.65	C_18_H_34_O_2_	As mentioned
40.46	Hexadecanoic acid and methyl ester	1.06	C_17_H_34_O_2_	Antioxidant, antimicrobial, and anti-inflammatory agents with compounds that decrease the effects of certain toxicants (Mazumder et al., 2020)
40.76	Quinindoline	1.01	C_18_H_14_N_2_	Antimicrobial, antifungal, antihelmintic, cardiotonic, anticonvulsant, and anti-inflammatory activities (Marella et al., 2013)
41.46	9-Octadecenoic acid (Z,Z)-, 2-hydroxy-1-(hydroxymethyl)ethyl ester	1.61	C_21_H_38_O_4_	Antioxidant, anti-inflammatory, antimicrobial, and diuretic activities (Muzahid et al., 2023)
44.73	9-Octadecenoic acid (Z)-	2.83	C_18_H_34_O_2_	As mentioned

^a^ Molecular formula, MF; peak area, PA; retention time, RT. Different chemicals’ biological activities were derived from the PubChem database (https://pubchem.ncbi.nlm.nih.gov/).

#### 3.1.2 Characterization of nano Sp

##### 3.1.2.1 Percentage yield, particle size, and zeta potential analyses

The percentage yield, EE, particle size, and zeta potential, and PDI were obtained.

The yield percentage was 23.37% ± 1.99% (Table 3) and EE was 93.17% ± 1.49%. Multiangle dynamic light scattering (MADLS) was used to analyze the nano Sp, revealing particle sizes below 300 nm (Table 3) with a mean value of 255.96 ± 38.21 nm. The average zeta potential was –29.47 ± 8.31 mV, and the PDI was 0.51.

**TABLE 3:** Polymeric nano Sp characteristics.

Parameter	Mean	Range
% Yield	23.37 ± 1.99	21.15–25.0
% EE	93.17 ± 1.49	91.18–94.68
Particle size (nm)	255.96 ± 38.21	218.4–294.8
Zeta potential (mV)	−29.47 ± 8.31	−19.99 to −35.52
PDI	0.51 ± 0.11	0.4–0.62

The following formulae can be used to determine EE:

%EE=− (total drug conc.−supernatant drug conc./total drug conc.×100%.

##### 3.1.2.2 SEM

**FIGURE 2:**
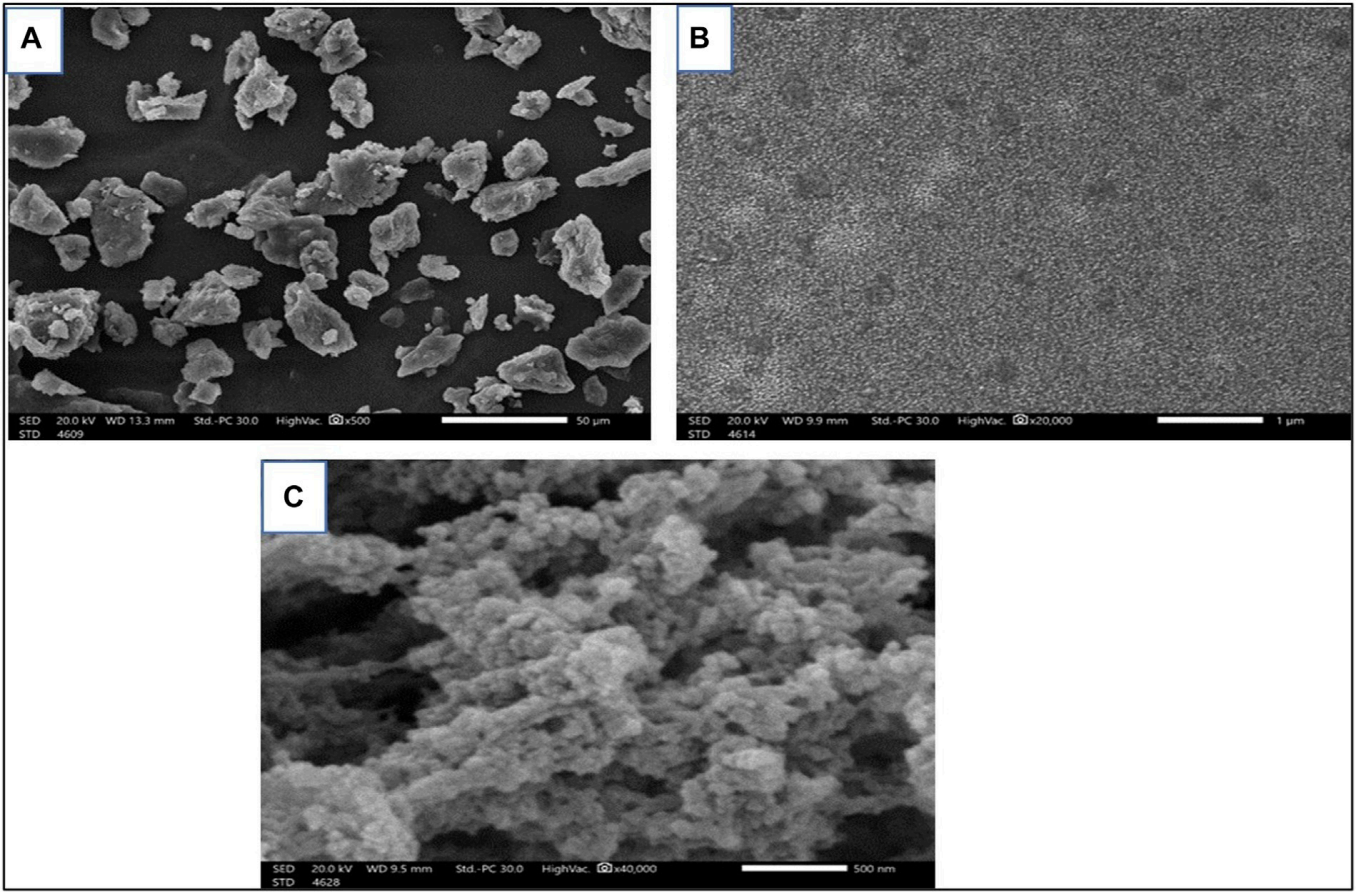
SEM images of **(A)** free Sp and **(B, C)** different magnifications of nano Sp.

Figure 2 shows how SEM was used to examine the particle morphologies, sizes, and shapes of free Sp and nano Sp. Figure 2A (magnification: ×500) illustrates that the free Sp particles are grouped unevenly and have irregular shapes and sizes; the size cannot be estimated because of the irregular shapes. Nano Sp appeared as spherical clusters (magnification: ×20,000 and ×40,000) that were almost identical in size (less than 30 nm) (Figures 2B, C).

##### 3.1.2.3 FTIR examination

**FIGURE 3:**
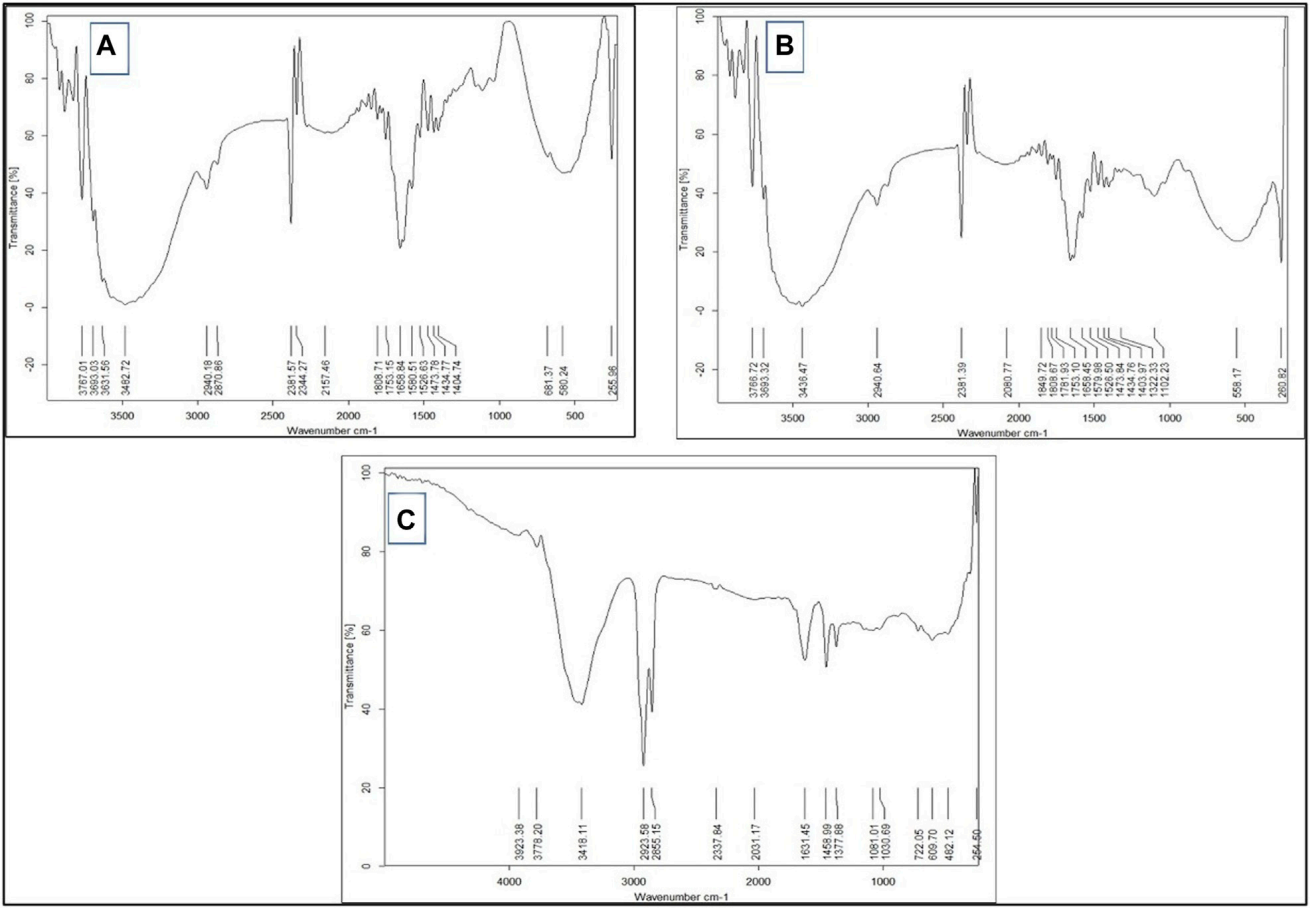
FTIR spectra of **(A)** free Sp, **(B)** nano Sp, and **(C)** chitosan.

The FTIR spectra of the free Sp and nano Sp are shown in Figure 3. The spectrum of free Sp (Figure 3A) shows a characteristic peak at −3,473.7 cm^−1^, which represents the hydroxyl group (OH) stretching vibration; another peak at −2,943.29 cm^−1^ indicates the alkane CH stretching. A sharp narrow peak at −2,382.02 cm^−1^ was attributed to the stretching vibrations of the alkyne groups; the peak at −1,660.66 cm^−1^ was attributed to the stretching vibrations of the alkene groups, and the peak at −1,581.58 cm^−1^ was due to the asymmetric stretching of the nitro compounds. The strong and broad peak in the fingerprint area at −577.41 cm^−1^ represents the stretching vibrations of the alkyl halides. Furthermore, the vibrations of the amide grouping was detected at 1,581.58 cm^–1^, which characterizes the protein nature of the microalgae (Theivandran et al., 2015). The nano Sp spectrum is almost identical to that of the free Sp; however, its % transmittance was lower (Table 4). The FTIR spectrum of nano Sp (Figure 3B) shows minor differences in the corresponding peak strengths at −2,382.02 cm^–1^, –1,581.58 cm^–1^, and –577.41 cm^–1^ when compared to that of free Sp (Doshi et al., 2007; Djelal et al., 2010). Figure 3C shows the FTIR spectrum of chitosan.

**TABLE 4:** FTIR analyses of free Sp and nano Sp.

	Frequency	Functional group	% Transmission of free Sp	% Transmission of nano Sp
1	−2,382.02 cm^1^	Stretching vibrations of alkyne groups	33.25	28.53
2	−1,581.58 cm^1^	Asymmetric stretching of nitro compounds	25.448	22.347
3	−1,116.75	C-N stretching vibrations of aliphatic amines	78.398	42.941
4	−577.41 cm^1^	Stretching vibrations of alkyl halides	50.977	28.077

### 3.2 *In vivo* experiments

#### 3.2.1 Impacts of different treatments on cardiac enzymes

As shown in Table 5, the Cyclo group exhibited remarkable increases of 155.49%, 105.74%, 451.76%, and 826.07% in the serum levels of SGOT, LDH, CK-MB, and cTnI enzyme activities, respectively, compared to the control; conversely, rats pretreated with TEL demonstrated substantial decreases of 36.05%, 30.03%, 42.36%, and 58.38% of the cardiac enzymes, significant decreases of 24.77%, 19.49%, 27.2%, and 30.84% of the cardiac enzymes following pretreatment with free Sp, and considerable reductions of 44.02%, 41.28%, 74.06%, and 67.85% of the cardiac enzyme levels after treatment with Nano Sp, respectively, when compared to the Cyclo Gp. In addition, pretreatment with nano Sp substantially decreased the cardiac enzymes by 12.47%, 16.08%, 54.99%, and 22.75% as compared with the 25.29%, 27.07%, 64.37%, and 53.51% values by TEL, respectively, compared to the free Sp group.

**TABLE 5:** Impacts of different treatments on serum levels of cardiac enzymes.

Group	SGOT (U/L)	LDH (U/L)	CK-MB (U/L)	cTnI (pg/mL)
VC	67.32 ± 2.930	435.2 ± 9.364	84.62 ± 6.319	34.9 ± 8.33
PC	72.71 ± 3.101	443.9 ± 19.99	84.96 ± 7.962	45.66 ± 7.811
TEL	69.91 ± 3.942	441.2 ± 7.236	88.96 ± 6.017	42.34 ± 7.42
Free Sp	72.15 ± 1.650	443.1 ± 7.648	87.97 ± 7.55	43.84 ± 8.71
Nano Sp	71.44 ± 1.578	450.9 ± 6.019	78.17 ± 9.29	41.84 ± 6.89
Cyclo	172 ± 7.069^*^	895.4 ± 18.81^*^	466.9 ± 12.26^*^	323.2 ± 8.489^*^
TEL + Cyclo	110 ± 5.332^*a^	626.5 ± 24^*a^	269.1 ± 23.16^*a^	134.5 ± 8.753^*a^
Free Sp + Cyclo	129.4 ± 6.924^*ab^	720.8 ± 12.36^*ab^	339.9 ± 8.39^*ab^	223.5 ± 8.572^*ab^
Nano Sp + Cyclo	96.28 ± 4.64^*abc^	525.7 ± 13.07^*abc^	121.1 ± 10.41^*abc^	103.9 ± 8.325^*abc^

Values were recorded as mean ± SD (n = 6). * means significant vs. control group.

^a^ Means significant vs. Cyclo group.

^b^ Means significant vs. TEL + Cyclo group.

^c^ Means significant vs. free Sp + Cyclo group. Cyclo, cyclophosphamide; Sp, *Spirulina platenesis* extract; nano Sp, nanoformulated Sp extract. *p* ≤ 0.05.

#### 3.2.2 Impacts of different treatments on ROS biomarkers in cardiac tissues

**FIGURE 4:**
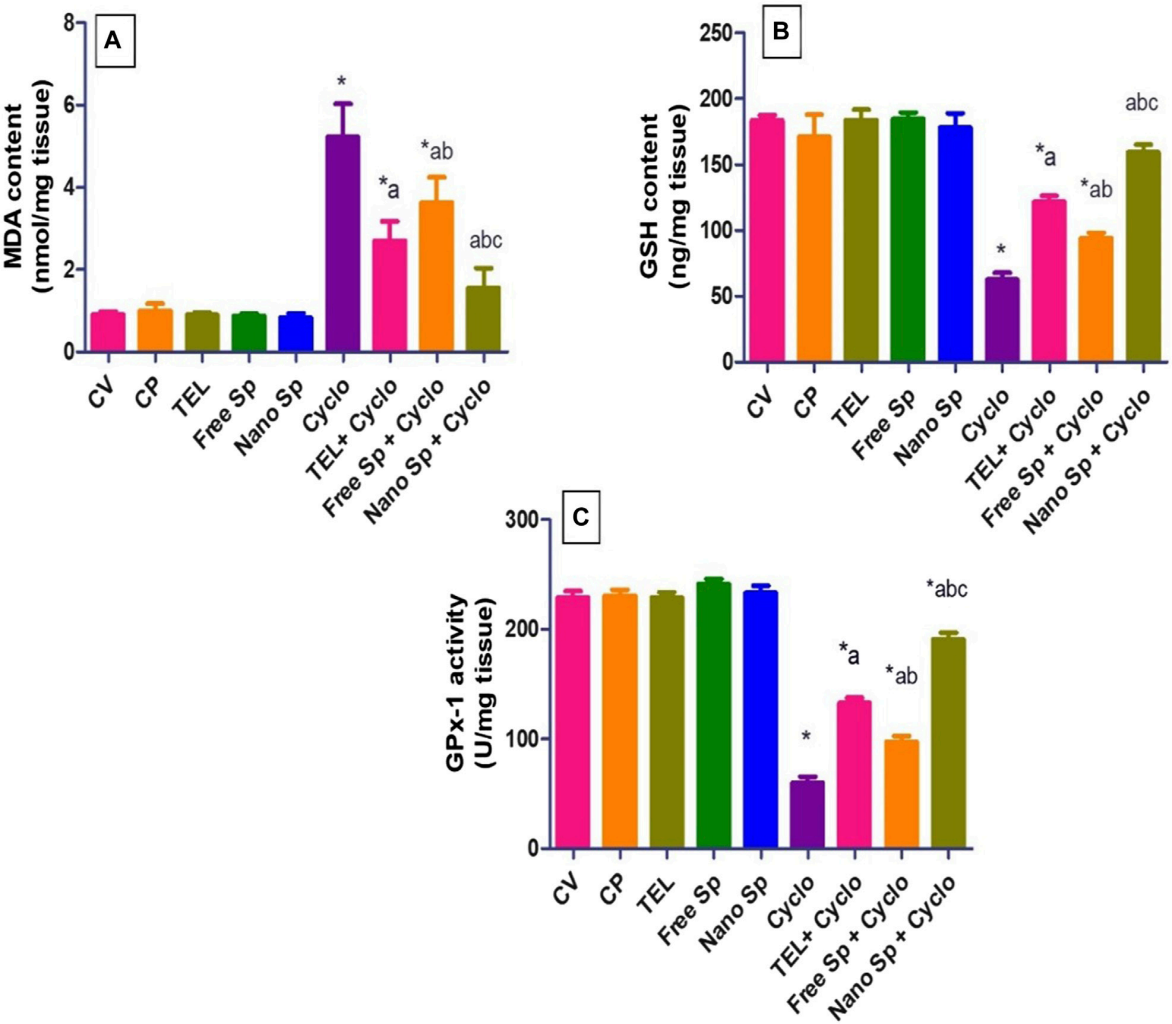
Impacts of different treatments on ROS biomarkers in cardiac tissues: **(A)** MDA content, **(B)** GSH content, and **(C)** GPX-1 enzyme activity. The values were recorded as mean ± SD (*n* = 6). * means significant vs. control group, ^a^ means significant vs. Cyclo group, ^b^ means significant vs. TEL + Cyclo group, and ^c^ means significant vs. free Sp + Cyclo group. Cyclo: cyclophosphamide, Sp: *Spirulina platenesis* extract, nano Sp: nanoformulated Sp extract. *p* ≤ 0.05.

It is seen from Figure 4 that the Cyclo group exhibits a considerable increase in MDA (474.89%) compared to the control. Conversely, pretreatments with TEL, free Sp, and nano Sp revealed substantial reductions of 48.08%, 30.45%, and 70.31% in MDA as compared to the Cyclo treatment group. Furthermore, animals pretreated with nano Sp demonstrated substantial reductions of 42.8% and 57.32% in MDA content than the rats treated with TEL and free Sp, respectively.

Similarly, GSH content in the Cyclo group was substantially lower 65.94% than that in the control group. Furthermore, the depletion of cardiac GSH content was returned by pretreatment with TEL, free Sp, and nano Sp (94.61%, 50.65%, and 155.23%, respectively) in comparison to the Cyclo group. Pretreatment with nano Sp also demonstrated remarkable increases of 31.14% and 69.42% in the GSH content than the TEL and free Sp groups, respectively.

The results show that GPX-1 enzyme activity in the Cyclo group was substantially reduced (73.85%) in comparison to that in the control. The depleted activity could be reversed by pretreatment with TEL, free Sp, and nano Sp (122.33%, 62.77%, and 218.62%, respectively) in comparison to the Cyclo group. The animals pretreated with nano Sp exhibited considerable increments of 26.78% and 95.75% in this enzyme activity than the TEL and free Sp groups, respectively.

#### 3.2.3 Impacts of different treatments on p-AKT in cardiac tissues

**FIGURE 5:**
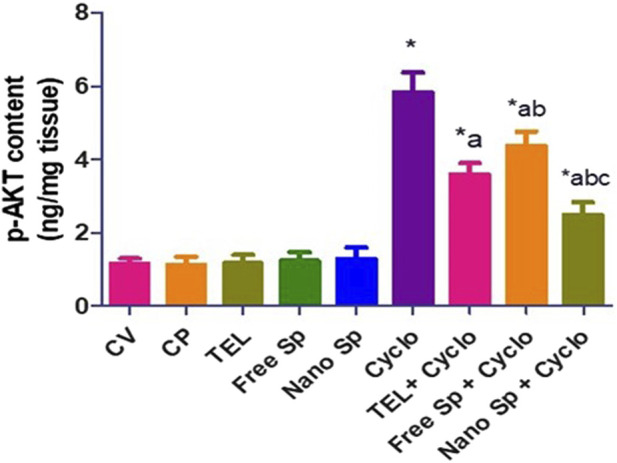
Impacts of different treatments on p-AKT content in cardiac tissues. The values were recorded as mean ± SD (*n* = 6). * means significant vs. control group, ^a^ means significant vs. Cyclo group, ^b^ means significant vs. TEL + Cyclo group, and ^c^ means significant vs. free Sp + Cyclo group. Cyclo: cyclophosphamide, Sp: *Spirulina platenesis* extract, nano Sp: nanoformulated Sp extract. *p* ≤ 0.05.

Figure 5 shows that the Cyclo group exhibits a remarkable increase in p-AKT (411.4%) content in relation to the control. On the other hand, pretreatments with TEL, free Sp, and nano Sp demonstrate remarkable reductions of 38.42%, 25.04%, and 57.2%, respectively, of p-AKT content in relation to the Cyclo group. In addition, animals pretreated with nano Sp display substantial declines of 30.64% and 43.02% in p-AKT content than those treated with TEL and free Sp, respectively.

#### 3.2.4 Impacts of different treatments on apoptotic gene expressions (*Bax* and *Bcl2*)

**FIGURE 6:**
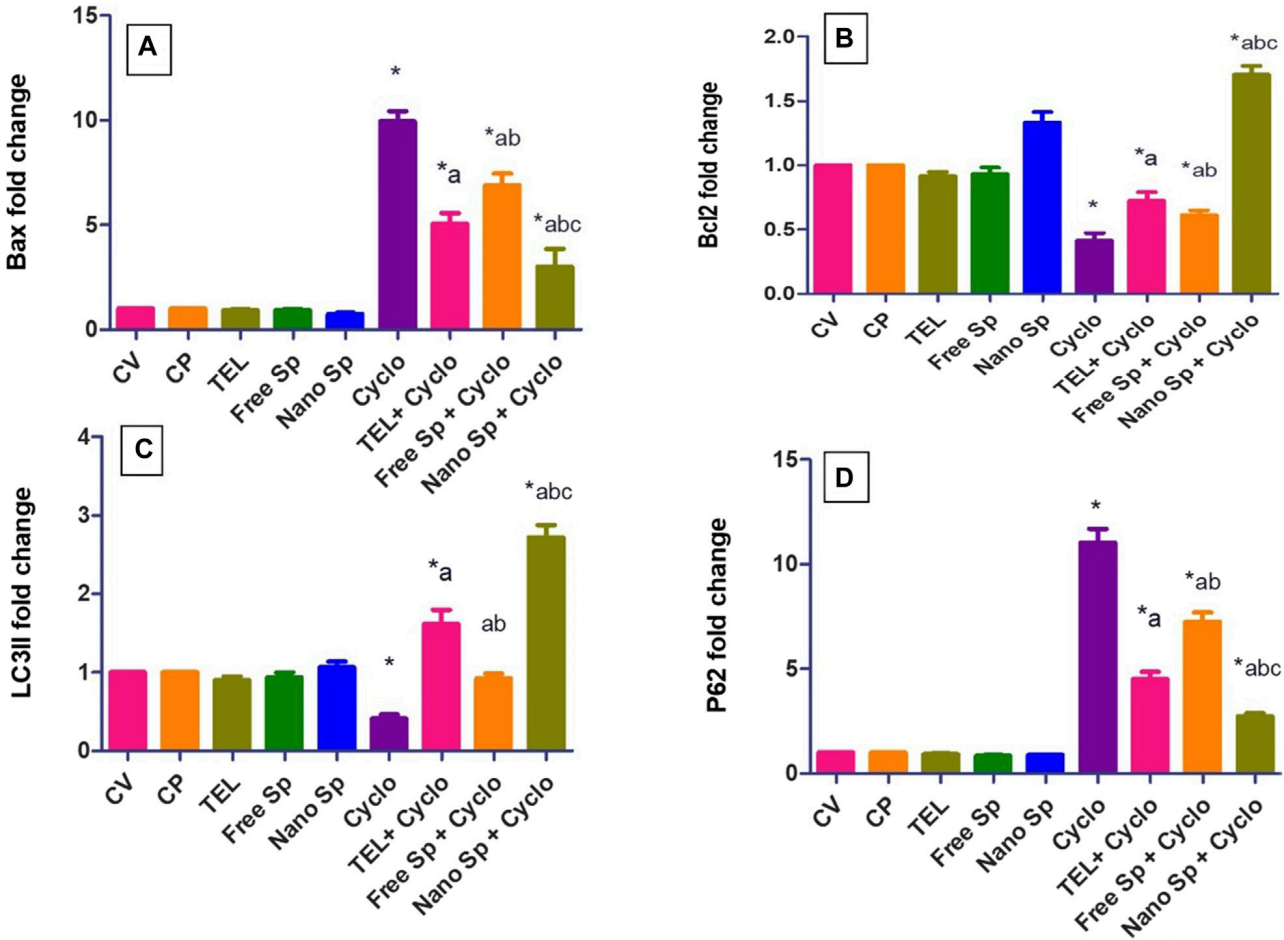
Impacts of different treatments on various gene expressions in cardiac tissues: **(A)** Bax, **(B)** Bcl2, **(C)** LC3II, and **(D)** p62 gene expressions. The values were recorded as mean ± SD (*n* = 6). * means significant vs. control group, ^a^ means significant vs. Cyclo group, ^b^ means significant vs. TEL + Cyclo group, and ^c^ means significant vs. free Sp + Cyclo group. Cyclo: cyclophosphamide, Sp: *Spirulina platensis* extract, nano Sp: nanoformulated Sp extract. *p* ≤ 0.05.

Figure 6 shows that the Cyclo group displays remarkable upregulation in the cardiac level of Bax gene expression (896%) in relation to the control. Conversely, the TEL, free Sp, and nano Sp groups display substantial downregulations of 41.16%, 30.82%, and 69.86%, respectively, in Bax gene expressions than the Cyclo group. Moreover, nano Sp pretreatment exhibited substantial downregulations of 40.67% and 56.43% in the cardiac levels of Bax gene expression than TEL and free Sp, respectively.

The Cyclo group also reveals a significant downregulation (58.7%) in the cardiac level of Bcl2 gene expression than the control. However, TEL, free Sp, and nano Sp treatments show considerable upregulations of 75.3%, 48.62%, and 314.07% in Bcl2 gene expressions, respectively, than the Cyclo group. In addition, the nano Sp group exhibits substantial upregulations of 136.5% and 178.5% in the cardiac levels of Bcl2 gene expression than TEL and free Sp, respectively.

#### 3.2.5 Impacts of different treatments on autophagic gene expressions (*LC3II* and *p62*)

Figure 6 shows that the Cyclo group displays a substantial downregulation (59%) in the cardiac level of LC3II gene expression in relation to the control. Pretreatments with TEL, free Sp, and nano Sp display substantial upregulations of 294.63%, 125.12%, and 564.14%, respectively, in LC3II gene expressions than the Cyclo group. In addition, rats pretreated with nano Sp exhibit remarkable upregulations of 69.13% and 195% in the cardiac levels of LC3II gene expression than TEL and free Sp, respectively.

Conversely, the Cyclo group displays a substantial upregulation (1003%) in the cardiac level of p62 gene expression in relation to the control. However, pretreatments with TEL, free Sp, and nano Sp display considerable downregulations of 59.11%, 34.36%, and 75.28%, respectively, in their p62 gene expressions than the Cyclo group. Furthermore, animals pretreated with nano Sp exhibit substantial downregulations of 39.68% and 62.45% in their cardiac levels of p62 gene expression than TEL and free Sp, respectively.

#### 3.2.6 Impacts of different treatments on *PON-1* gene expression

**FIGURE 7:**
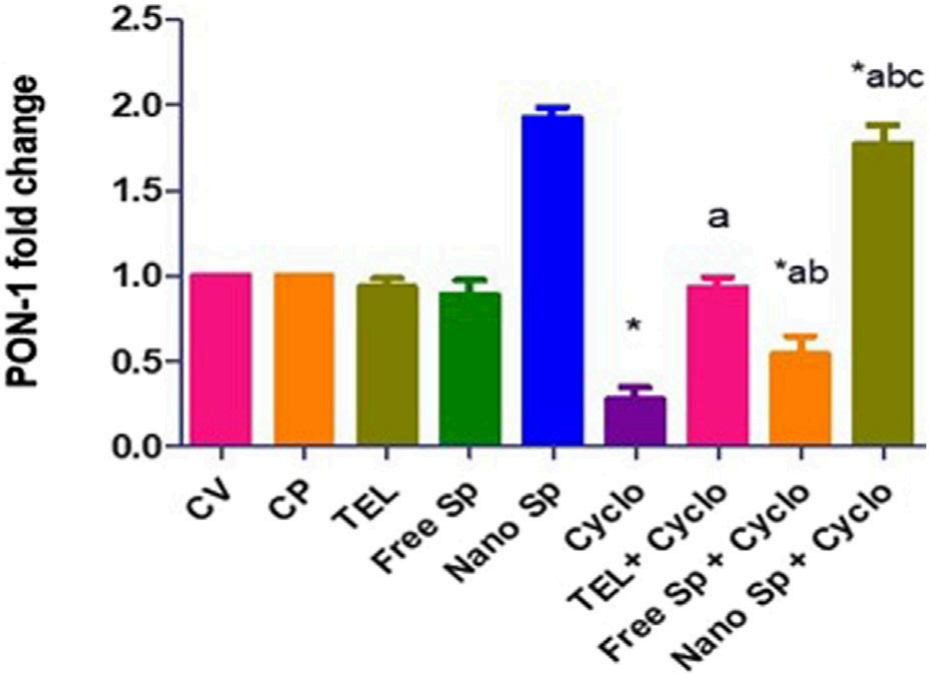
Impacts of different treatments on PON-1 gene expression in cardiac tissues. The values were recorded as mean ± SD (*n* = 6). * means significant vs. control group, ^a^ means significant vs. Cyclo group, ^b^ means significant vs. TEL + Cyclo group, and ^c^ means significant vs. free Sp + Cyclo group. Cyclo: cyclophosphamide, Sp: *Spirulina platenesis* extract, nano Sp: nanoformulated Sp extract. *p* ≤ 0.05.

Figure 7 shows that the Cyclo group displays a remarkable downregulation (72.2%) in the cardiac level of PON-1 gene expression in relation to the control. Pretreatments with TEL, free Sp, and nano Sp display substantial upregulations of 234.5%, 95.32%, and 536.69%, respectively, in PON-1 gene expressions than the Cyclo group. Rats pretreated with nano Sp also display remarkable upregulations of 90.3% and 225.9% in the cardiac levels of PON-1 gene expression than TEL and free Sp, respectively.

#### 3.2.7 Impacts of different treatments on Cyclo-induced myocardial histopathological alterations

**FIGURE 8:**
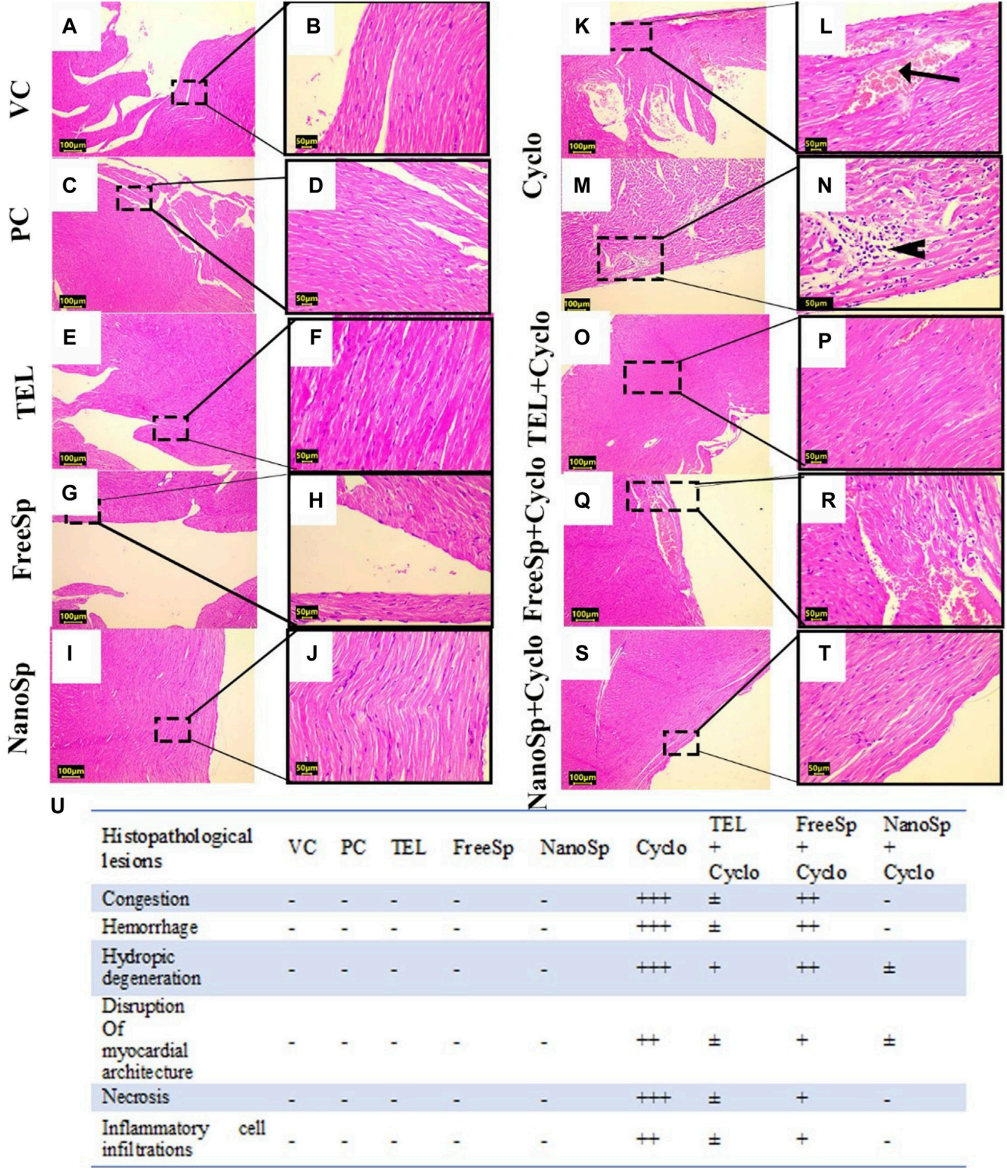
Impacts of different treatments on Cyclo-induced myocardial histopathological alterations: **(A, B)** vehicle control (VC), **(C, D)** polymer control (PC), **(E, F)** TEL, **(G, H)** free Sp, and **(I, J)** nano Sp showing typical myocardial histoarchitectures. **(K–N)** Cyclo-treated group showing vascular congestion (arrow) and myocardial necrosis with interstitial mononuclear inflammatory infiltrates (arrowhead). **(O, P)** TEL + Cyclo-treated group shows nearly normal histoarchitecture with mild congestion; **(Q, R)** free Sp + Cyclo-treated group shows moderate vascular congestion with mild interstitial inflammatory infiltrates; **(S, T)** nano Sp + Cyclo-treated group shows nearly normal myocardial histoarchitecture. **(U)** The scoring system is a scale ranging from negative (representing normal findings) to positive (indicating varying degrees of abnormality), including borderline ±, mild +, moderate ++, and severe levels +++. Scale bar = 100 µm and 50 µm. Hematoxylin and eosin (H&E).

As illustrated in Figure 8, the cardiac tissues from the PC, VC, TEL, free Sp, and nano Sp rats show normal cardiac histoarchitecture with typical cross striations (Figures 8A–J). Animals subjected to Cyclo treatment exhibit substantial myocardial abnormalities represented by vascular congestion, sarcoplasmic vacuolation, deep acidophilic sarcoplasmic staining, multifocal separation of myocardial fibers with loss of transverse sarcoplasmic striations, interstitial edema, and hemorrhage (Figures 8K, L). The necrotic cardiomyocytes exhibit nuclear karyorrhexes and karyolysis with interstitial mononuclear inflammatory infiltrates (Figures 8M, N). Rats cotreated with Cyclo and free Sp show minimal myocardial lesions limited to moderate vascular congestion and mild mononuclear inflammatory infiltrates between the myofibrils (Figures 8Q, R). Rats receiving cotreatments of Cyclo with TEL (Figures 8O, P) and nano Sp (Figures 8S, T) show improvements in their myocardial histoarchitecture, as evidenced by the typical arrangement of cardiac myofibrils with centrally located nuclei and prominent nucleoli. This effect was more prominent in the nano Sp + Cylo treatment group.

#### 3.2.8 Impacts of different treatments on eNOS, caspase-3, p62, and beclin-1 immunoreactivities in Cyclo-induced myocardial injury

**FIGURE 9:**
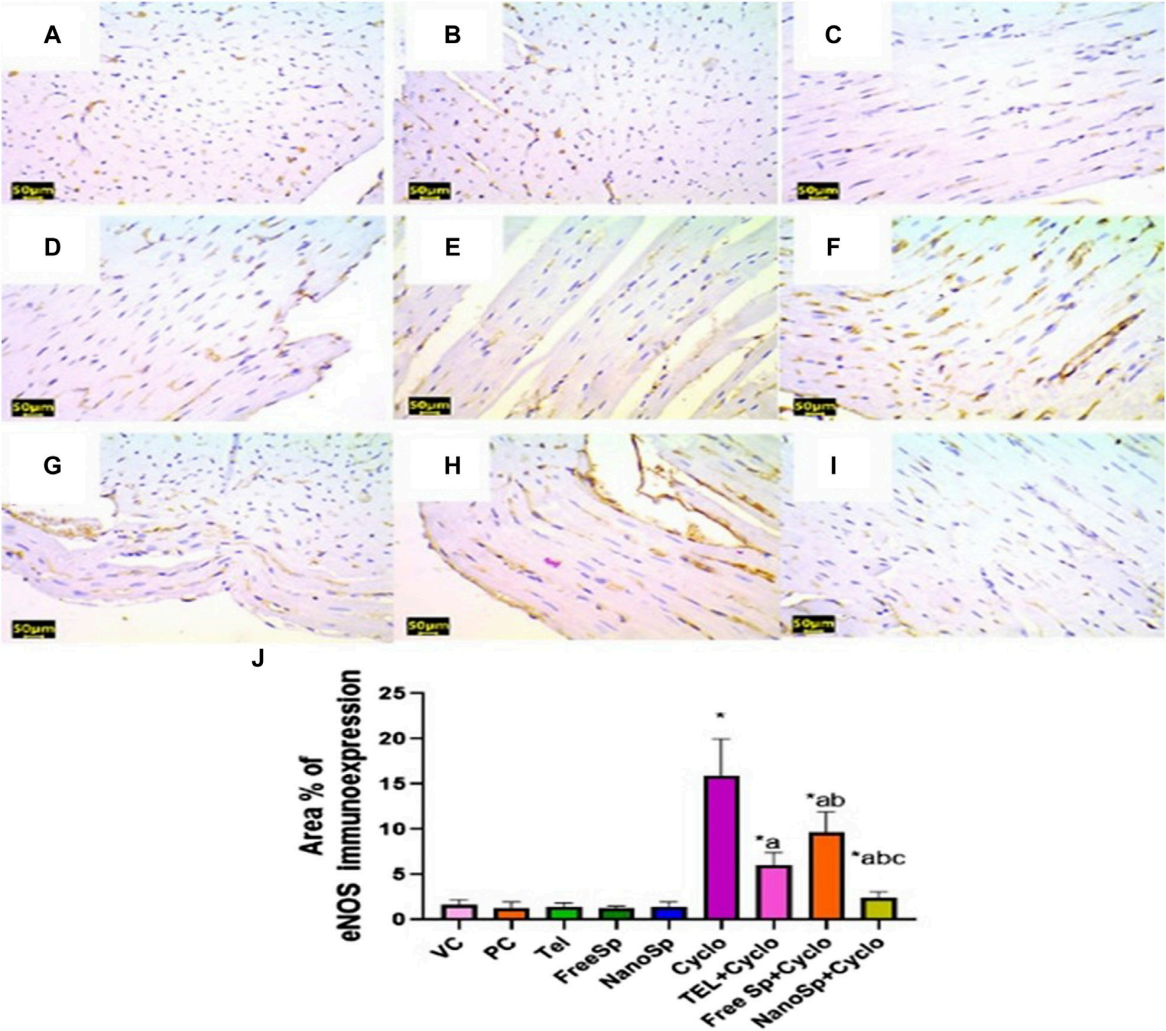
Impacts of different treatments on eNOS immunoreactivity in Cyclo-induced myocardial injury: **(A)** vehicle control (VC), **(B)** polymer control (PC), **(C)** TEL, **(D)** free Sp, and **(E)** nano Sp showing weak eNOS immunoreactivity. **(F)** Cyclo-treated group with nano Sp showing marked eNOS immunoreactivity. **(G)** TEL + Cyclo-treated group with nano Sp showing mild eNOS immunoreactivity. **(H)** Free Sp + Cyclo-treated group with nano Sp showing moderate eNOS immunoreactivity. **(I)** Nano Sp + Cyclo-treated group with nano Sp showing weak eNOS immunoreactivity. **(J)** Semiquantitative statistical analyses for the percentage area of eNOS immunoreactivity. Scale bar = 50 µm. The values are expressed as mean ± SD. Endothelial nitric oxide synthase (eNOS) immunostain.

As illustrated in Figure 9, mild eNOS immunoexpressions are observed in the PC, VC, TEL, free Sp, and nano Sp groups; however, the administration of Cylo results in marked eNOS immunoexpressions, while Cyclo treatment with free Sp exhibits moderate immunoreactivity. Additionally, Cyclo treatments with TEL and nano Sp show weak eNOS immunoexpressions. Semiquantitative statistical analyses did not show any significant differences in the eNOS immunoexpressions among the PC, VC, TEL, free Sp, and nano Sp groups. On the other hand, the Cyclo group revealed a significant increase in eNOS immunoexpression (829%) than the control. Furthermore, Cyclo-intoxicated rats with TEL, free Sp, and nano Sp exhibited considerable downregulations in their eNOS immunoexpressions (61.93%, 39.04%, and 84.94%, respectively) as compared with the Cyclo group. Furthermore, rats treated with nano Sp reveal considerable downregulations of 60.43% and 75.30% in their eNOS immunoexpressions than TEL and free Sp, respectively (Figure 9J).

**FIGURE 10:**
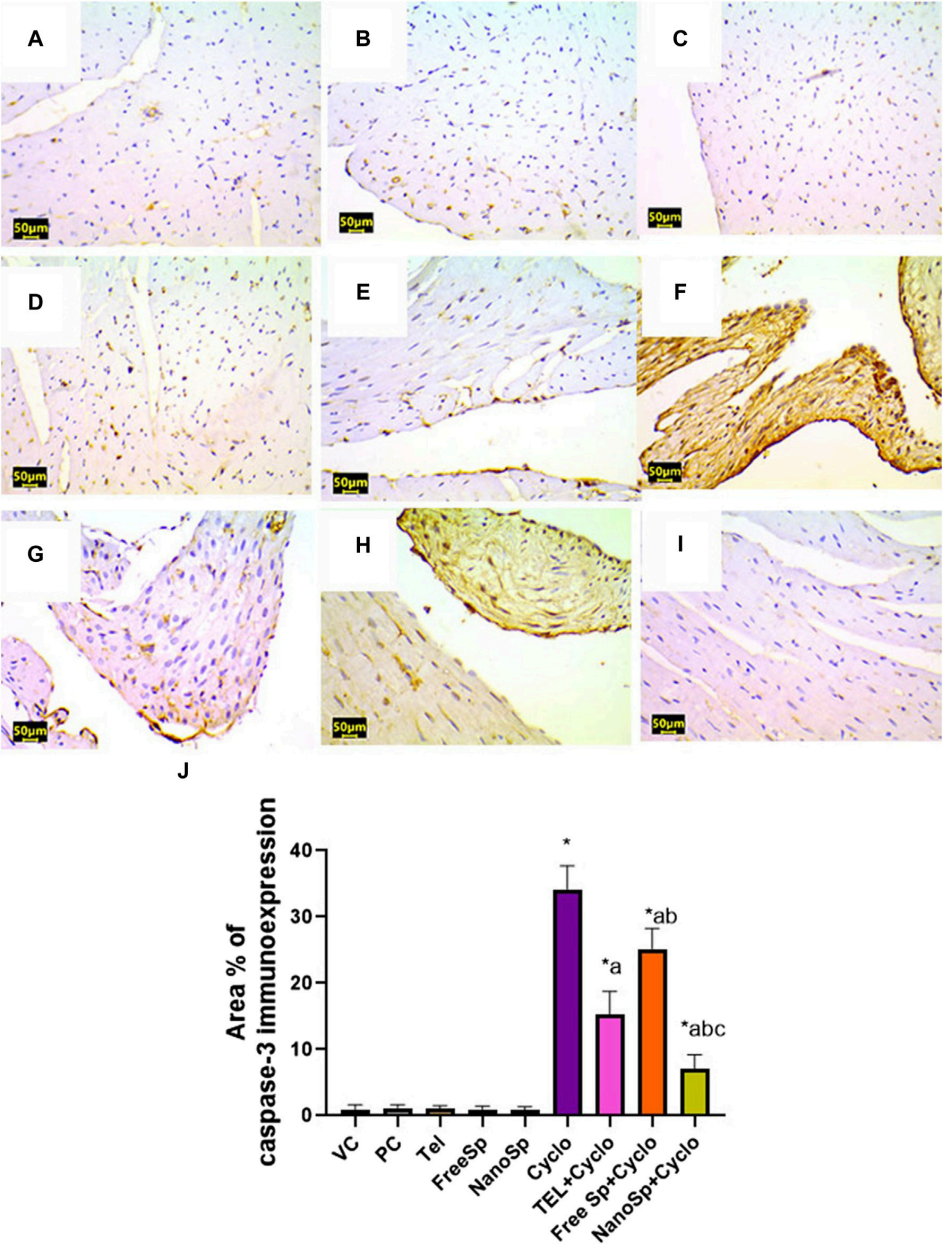
Impacts of different treatments on caspase-3 immunoreactivity in Cyclo-induced myocardial injury: **(A)** vehicle control (VC), **(B)** polymer control (PC), **(C)** TEL, **(D)** free Sp, and **(E)** nano Sp showing negative caspase-3 immunoreactivity. **(F)** Cyclo-treated group showing marked caspase-3 immunoreactivity. **(G)** TEL + Cyclo-treated group showed weak caspase-3 immunoreactivity. **(H)** Free Sp + Cyclo-treated group showing moderate caspase-3 immunoreactivity. **(I)** Nano Sp + Cyclo-treated group showing nearly negative caspase-3 immunoreactivity. **(J)** Semiquantitative statistical analysis for the percentage area of caspase-3 immunoreactivity. Scale bar = 50 µm. The values are expressed as mean ± SD.

**FIGURE 11:**
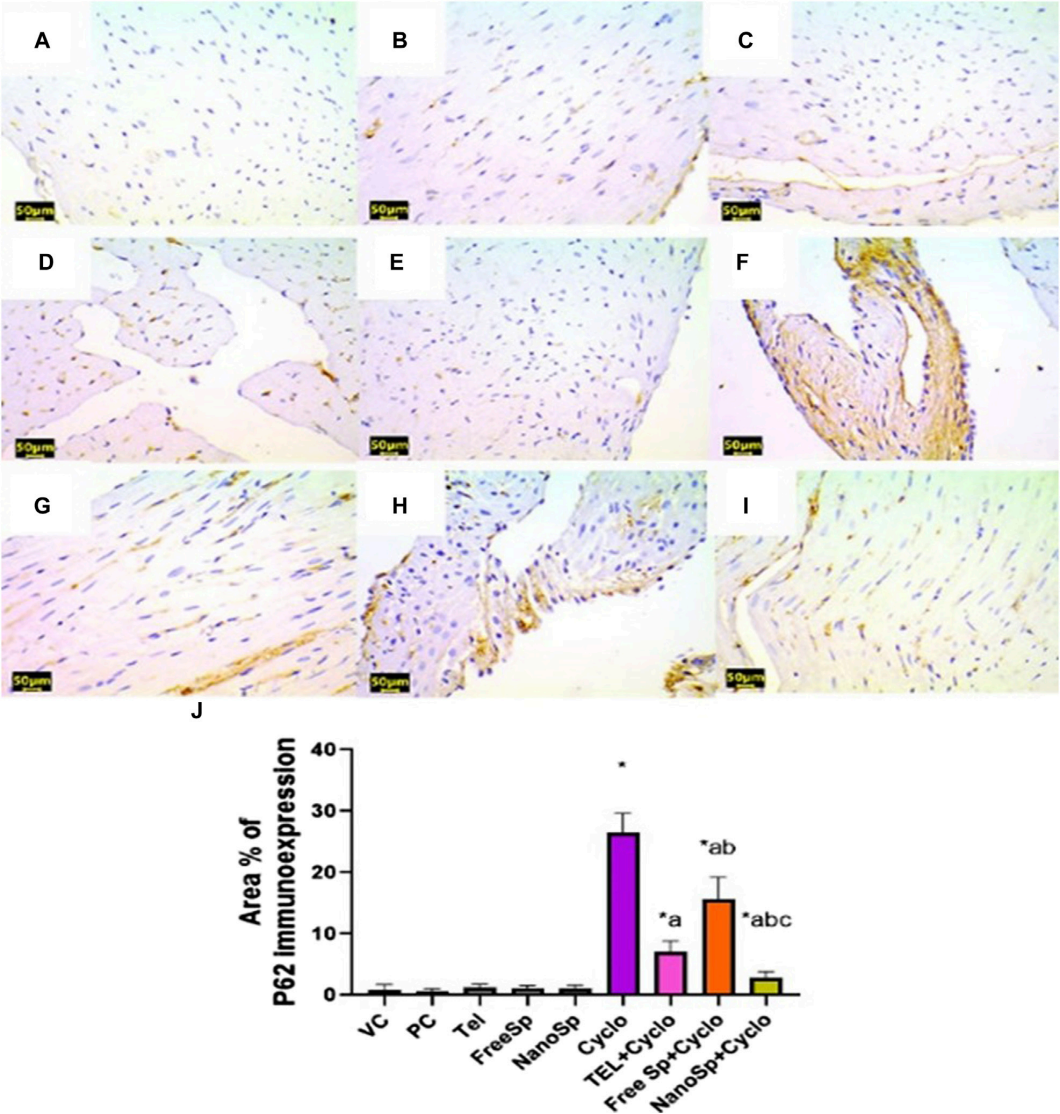
Impacts of different treatments on p62 immunoreactivity in Cyclo-induced myocardial injury: **(A)** vehicle control (VC), **(B)** polymer control (PC), **(C)** TEL, **(D)** free Sp, and **(E)** nano Sp showing negative p62 immunoreactivity. **(F)** Cyclo-treated group showing marked p62 immunoreactivity. **(G)** TEL + Cyclo-treated group showing mild p62 immunoreactivity. **(H)** Free Sp + Cyclo-treated group showing moderate p62 immunoreactivity. **(I)** Nano Sp + Cyclo-treated group showing little p62 immunoreactivity. **(J)** Semiquantitative statistical analysis for the percentage area of p62 immunoreactivity. Scale bar = 50 µm. The values are expressed as mean ± SD.

As illustrated in Figures 10, 11, negative immunoexpressions are observed for caspase-3 and p62 in the PC, VC, TEL, free Sp, and nano Sp groups; however, the administration of Cylo results in intense caspase-3 and p62 immunoexpressions, while Cyclo treatment with free Sp exhibits moderate immunoreactivity. Additionally, Cyclo treatments with TEL and nano Sp show mild-to-negative caspase-3 and p62 immunoexpressions. Semiquantitative statistical analyses show no significant differences in caspase-3 and p62 immunoexpressions among the PC, VC, TEL, free Sp, and nano Sp groups. On the other hand, the Cyclo group revealed significant upregulations in caspase-3 (3,489%) and p62 (2578%) immunoexpressions as compared to the control animals. Furthermore, Cyclo treatments with TEL, free Sp, and nano Sp show significant downregulations in caspase-3 (55.34%, 26.28%, and 79.44%, respectively) and p62 (73.32%, 40.66%, and 88.76%, respectively) immunoexpressions relative to the Cyclo group. Rats treated with nano Sp also exhibit substantial downregulations of 53.96% and 57.87% in the cardiac levels of caspase-3 and p62 immunoexpressions than TEL as well as considerable downregulations of 65.07% and 81.05% than the free Sp group, respectively (Figures 10, 11J).

**FIGURE 12:**
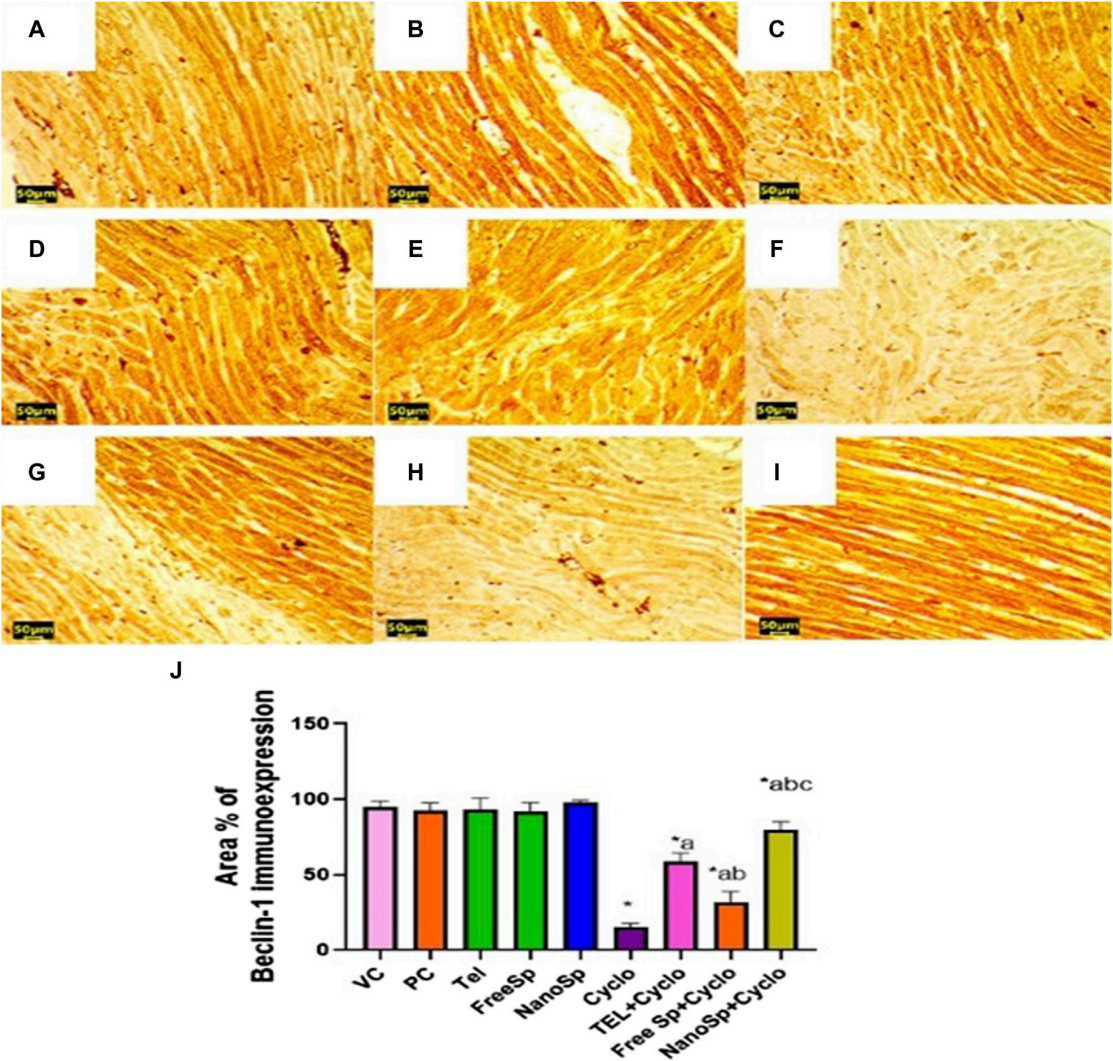
Impacts of different treatments on beclin-1 immunoreactivity in Cyclo-induced myocardial injury: **(A)** vehicle control (VC), **(B)** polymer control (PC), **(C)** TEL, **(D)** free Sp, and **(E)** nano Sp showing intense beclin-1 immunoreactivity. **(F)** Cyclo-treated group showing weak beclin-1 immunoreactivity. **(G)** TEL + Cyclo-treated group showing marked beclin-1 immunoreactivity. **(H)** Free Sp + Cyclo-treated group showing moderate beclin-1 immunoreactivity. **(I)** Nano Sp + Cyclo-treated group showing intense beclin-1 immunoreactivity. **(J)** Semiquantitative statistical analysis for the percentage area of beclin-1 immunoreactivity. Scale bar = 50 µm. The values are expressed as mean ± SD.

As illustrated in Figure 12, moderate beclin-1 immunoexpressions are observed in the PC, VC, TEL, free Sp, and nano Sp groups; however, the administration of Cylo shows mild-to-negative beclin-1 immunoexpression, while Cyclo treatment with free Sp exhibits moderate immunoreactivity. Additionally, Cyclo treatments with TEL and nano Sp show marked beclin-1 immunoexpressions. Semiquantitative statistical analyses show no significant differences in beclin-1 among the PC, VC, TEL, free Sp, and nano Sp groups. On the other hand, Cyclo-intoxicated rats reveal a significant upregulation in beclin-1 (84.09%) immunoexpression relative to the control. Furthermore, Cyclo treatments with TEL, free Sp, and nano Sp reveal considerable downregulations in the beclin-1 immunoexpressions (289%, 109%, and 431%, respectively) relative to the Cyclo group. The Nano Sp + Cylo group also exhibited considerable increases of 36.42% and 153.4% in cardiac beclin-1 immunoexpressions than the TEL and free Sp groups, respectively (Figure 12J).

## 4 Discussion

Cyclophosphamide is a cardiotoxic agent that induces direct myocardial endothelial damage and destruction of the myocardial cells. As noted by Tripathi and Jena (2009), Cyclo is a chemotherapeutic agent that inhibits cell division by damaging the DNA. Alkylating substances such as 4-hydroxy cyclophosphamide, aldophosphamide, mustard, and acrolein are produced during the metabolism of Cyclo. Of these, acrolein is a dangerous chemical that interacts with the proteins, membrane lipids, and DNA in the body (Zanchi et al., 2015); hence, there is a need for a transformative approach in the management of its side effects to enhance the quality of life for patients. Exploring new strategies could offer valuable insights into designing and developing various compounds with potential protective properties. *Spirulina platensis* has been advocated as a safe food for human use by several investigators (Karkos et al., 2011). Therefore, the purpose of this work was to identify the bioactive components in Sp methanolic extract and synthesize Sp methanolic extract/chitosan nanoparticles using the ionic gelation technique. Additionally, the protective efficacies of TEL and Sp nanoparticles were ascertained against cardiotoxicity caused by Cyclo.

*Spirulina* and its primary ingredients like C-phycocyanin, fatty acids, and phenolic and flavonoid compounds have anti-inflammatory, neuroprotective, hepatoprotective, immunomodulatory, and anticancer properties (Reddy et al., 2000; Khan et al., 2005b). Given its apparent non-toxicity and significant multiorgan protection against a range of chemical and drug-induced toxic assaults, *spirulina* has attracted increasing interest (Zareba et al., 2007; Lu et al., 2010).

In this study, GC-MS examination of the Sp methanolic extract revealed the presence of several bioactive chemicals, including hexadecanoic acid (28.29%), 9,12-octadecenoic acid (Z) (22.29%), gamolenic acid (9.27%), palmitoleic acid (6.71%), heptadecane (6.44%), and 9-octadecenoic acid (Z) (4.48%). According to Alves et al. (2013), these substances could be the source of the extract’s antioxidant activities. Hexadecanoic acid and hexadecanoic acid methyl ester or palmitic acid, for instance, are fatty acids with antibacterial, anti-inflammatory, and antioxidant properties that may also be used to lessen the effects of certain hazardous substances (Asghar et al., 2011). Ganesh and Mohankumar (2017) reported that it is a precursor of several substances, including vitamins, phospholipids, glycolipids, and prostaglandins. Furthermore, 9,12-octadecadienoic acid (Z, Z) shows antibacterial and anti-inflammatory activities (Pinto et al., 2017). Phytol (PYT) is another diterpene that has been shown to exhibit anxiolytic, metabolism-regulating, antioxidant, antinociceptive, anti-inflammatory, immune-modulating, and antibacterial properties in many investigations (Islam et al., 2016; Zhang et al., 2016; Islam et al., 2018).

In the present study, chitosan was selected to encapsulate free Sp based on its advantage of being a natural biocompatible polymer (Zhang et al., 2006). Chitosan increases mucosal adhesion and the time required for permeation of the drug molecules; it also exhibits efflux pump inhibitory properties (Mikušová and Mikuš, 2021). As reported by Liang et al. (2017), absorption and bioavailability enhancements for tea phenols have been achieved with chitosan nanoparticles.

As reported in a previous study by Stanic-Vucinic et al. (2018), proteins constitute more than 60% of the content of free Sp; it was found that the greatest solubility of the protein content of free Sp was 59.6% ± 0.7% (w/w) at pH 10 (Benelhadj et al., 2016). Free Sp powder was levigated with glycerol and suspended using 1% tween 80 to enhance its solubility in deionized water at neutral pH. The application of low temperature (4°C) during sonication allows effective homogenization to prevent loss of the volatile components (Hamad et al., 2023). Perfect homogenization of the particles was also assured with a final sonication using an ice bath. The freeze-drying technique was selected to minimize loss of volatile components as much as possible (Nouri and Abbasi, 2018).

The zeta potential is an important factor in determining the stability of a nanoformulation (Zhang et al., 2008). A nanoformulation is stable if it has a high potential (exceeding +30 mV or below −30 mV) (Dhas et al., 2015). In the present study, the results show that a zeta potential of more than −20 mV reflects the stability of the formulation. Furthermore, MADLS provides improved measurement resolution compared to dynamic light scattering. The results demonstrate that about 27% of particles were in the range of 200 nm (Austin et al., 2020; Markova et al., 2022).

The SEM results show that nano Sp is a spherical entity with a particle size of approximately 30 nm. The FTIR spectra of free Sp and nano Sp were almost identical, especially in the fingerprint area, which illustrates that the nano Sp preserves almost all of the components in free Sp. The spectra were almost identical, except for slightly diminished transmittance that could be attributed to the interactions of the nitro components of free SP with the acidic groups of chitosan (Han, 2016).

The current investigations show that the Cyclo dose (200 mg/kg intraperitoneally) caused cardiotoxicity in the animal model. The serum SGOT, LDH, cTnI, and CK-MB levels increased in response to Cyclo treatment. Furthermore, the GSH content and GPx-1 enzyme activity are greatly reduced. These results are similar to those reported in literature (Ashry et al., 2013; MacAllister et al., 2013; Walsh et al., 2014). The observed data are consistent with the results presented by Attia et al. (2023), who showed that the administration of 200 mg/kg of Cyclo resulted in a substantial increment in the cardiac enzyme serum levels (cTnI, CK-MB, LDH, AST, and ALT) and degree of lipid peroxidation (LPO) in the rat model, in addition to the significant decreases in antioxidant enzymes (CAT, SOD, and GPx). The heightened levels of these enzymes are associated with myocardial damage, including myocardial necrosis, infarction, myocarditis, and heart failure (Subramaniam et al., 2013). The enzymes enclosed within the cardiomyocytes are released into the blood as a consequence of endothelial injury. The increase in LDH and CK levels in the cardiac tissues of the Cyclo-treated rats could potentially be attributed to the excessive generation of ROS, which in turn could lead to membrane damage by initiating the production of LPO and compromising the functionality and integrity of the membranes of the cardiomyocytes (Alhumaidha et al., 2016; Attia et al., 2023). In myocardial injury, the occurrence of oxidative stress associated with the mitochondria can potentially cause damage in the form of disintegration of the mitochondria and cellular necrosis. As a consequence of these events, the enzymes AST and ALT are subsequently liberated from the mitochondria into the bloodstream. It was noted that the administration of Cyclo led to notable augmentations in the functioning of the LDH and CK enzymes in the heart tissue relative to the control group; this increase was attributed to the considerable disintegration and formation of vacuoles in the cardiomyocytes. Furthermore, there was a complete absence of the cristae of the mitochondria in the heart tissue (Omole et al., 2018). The reductions of the serum levels of SGOT, LDH, cTnI, and CK-MB levels by TEL, free Sp, and nano Sp pretreatments were observed in this study. Iqbal et al. (2008) demonstrated that the administration of TEL significantly decreased the LDH and MDA levels while increasing GSH in doxorubicin-induced cardiotoxicity. Khan et al. (2005a) showed that Sp ameliorated doxorubicin-induced cardiotoxicity in mice models *via* downregulation of MDA and upregulation of SOD and GPx-1; this protective effect of Sp is attributed to its antioxidant constituents C-phycocyanin and β-carotene.

The PON-1 enzyme is a fascinating molecule attached to a high-density lipoprotein. It breaks down oxidized LDL cholesterol, preventing plaque buildup in the arteries. It also has anti-inflammatory properties to protect against heart disease (Camps et al., 2009; Costa et al., 2011). In this work, Cyclo induced reduction of PON-1 expression; however, increases in PON-1 expressions and restoration of the cardiac enzyme levels were noted in rats treated with TEL, free Sp, and nano Sp. This can be linked to favorable cardiovascular protection. These findings are consistent with literature (Ibrahim et al., 2019; Yousef et al., 2019) reporting the depletion of PON-1 in the plasma and heart tissues with long-term exposure to iron oxide, silver nanoparticles, and propolis. Dube et al. (2022) reported the protective effect of PON-1 on the heart in chronic renal disease; it has been shown that 40 mg/day of TEL treatment significantly upregulates PON-1 in both diabetic hypertensive and hypertensive patients (BARUTÇUOĞLU et al., 2010). Furthermore, Seyidoglu et al. (2021) reported that Sp supplementation upregulated PON-1 in a stress-induced rat model.

Cyclo-induced apoptosis is widely recognized as the principal process responsible for Cyclo-mediated cardiac toxicity because Cyclo initiates both the intrinsic and extrinsic apoptotic signals *via* diverse mechanisms (Ibrahim et al., 2023). Initiation of the apoptotic pathways can cause DNA fragmentation and chromatin condensation. The present findings demonstrate that the levels of Bax and caspase-3 increase following Cyclo injection while the level of BcL2 declines; this initiates the apoptotic pathway, which is associated with the presence of oxidative stress and subsequent DNA damage. Ultimately, these events culminate in the activation of the mitochondrial apoptotic pathway through a reduction in the levels of antiapoptotic proteins (Mahmoud et al., 2017; Ahmed et al., 2023). It has been demonstrated that 150 mg/kg/day of Cyclo markedly increase the proapoptotic proteins caspase-3 and caspase-9 in the cardiac tissues of Wistar rats (Adeyemi et al., 2024). Another study by Akram et al. (2024) demonstrated that a single intraperitoneal administration of Cyclo at a dose of 200 mg/kg significantly upregulates cleaved caspase-3 immunoexpression in the cardiac tissues of Swiss albino mice.

The administration of TEL, free Sp, and nano Sp to Cyclo-intoxicated rats resulted in significant upregulation of the BcL2 gene and considerable downregulations of the Bax gene and caspase-3 immunoexpression, illustrating that TEL, free Sp, and nano Sp may inhibit Cyclo-mediated apoptotic cell deaths in cardiomyocytes. Parallel to these results Arozal et al. (2010) showed that TEL has antiapoptotic properties against daunorubicin-induced cardiotoxicity, whereas TEL significantly decreases the number of TUNEL-positive nuclei and expression of activated caspase-7 in addition to upregulation of Bcl2 expression. Another experimental study by Elmorsi et al. (2023) showed that Sp monotherapy downregulated caspase-3 immunoreactivity in doxorubicin-induced cardiotoxicity. A study by Sinanoglu et al. (2012) reported that Sp supplementation (1000 mg/kg bw/day) for 7 days may be able to downregulate apoptosis in the kidneys of rats intoxicated with Cyclo through downregulation of caspase-3 expression. This is the first study that shows how nano Sp extract exerts antiapoptotic qualities.

Cyclo is regarded as the most prevalent chemotherapeutic agent that elicits vascular permeability and edema. It alters the structure and permeability of the endothelium in the blood vessels. Acrolein, a Cyclo metabolite, induces an elevation in the production of eNOS with subsequent formation of NO; NO possesses anti-inflammatory properties at the physiological level but has been implicated in inflammatory and oxidative pathways in abnormal circumstances (Papi et al., 2019). Additionally, Akt mediates the activation of eNOS.

Parallel to this phenomenon, this study shows that Cyclo induces an increase in the p-Akt content and eNOS protein expression, which are in line with the findings of previous reports (Sancho et al., 2014; Barberino et al., 2022; Elsayed et al., 2022). The reductions of p-Akt and eNOS by TEL, free Sp, and nano Sp pretreatments are observed in this study. Cho (2019) illustrated that the application of TEL substantially decreases the phosphorylation status of eNOS and NO within the aortic endothelium. An *in vitro* study showed that TEL decreased the phosphorylation status of Akt in human osteosarcoma cells (Wang and Wang, 2018). On the contrary, an *in vitro* study by Sibiya et al. (2022) showed that *Spirulina platensis* downregulated both Akt and eNOS expressions in HepG2 cells. To the best of the authors’ knowledge, this is the first study that tests the effects of TEL and nano Sp on Akt and eNOS expressions in Cyclo-induced cardiotoxicity.

In the same context, cell self-digestion or autophagy is a defensive mechanism for cells that might be triggered by a variety of stressors, including oxidative stress (Munemasa and Kitaoka, 2015). The results indicate that Cyclo is associated with inhibition of autophagy signals, as evidenced by the marked increment in p62 immunoexpression and downregulation of LC3II gene expression and beclin-1 immunoreactivity. Liu et al. (2020) showed that Cyclo increases LC3II/LC3I ratio and downregulates SQSTM1/p62 gene expression in the urinary bladder of the cystitis mouse model. Wang et al. (2017) showed that amplified Akt phosphorylation and mTOR led to a reduction in apoptosis levels but augmented the LC3II/LC3I ratio and number of cells experiencing autophagy.

Interestingly, the administration of TEL, free Sp, and nano Sp to Cyclo-intoxicated rats resulted in significant downregulation of p62 immunoreactivity and considerable increments in LC3II gene expression and beclin-1 immunoreactivity with increased p-AKT content, illustrating that TEL, free Sp, and nano Sp can activate autophagy flux in Cyclo-treated rats. To the best of the authors’ knowledge, nano Sp extract showed remarkable protection against cardiotoxicity induced by Cyclo compared with free Sp or TEL treatment via modulations of the oxidative stress, apoptosis, and autophagy pathways.

## 5 Conclusion

Oxidative stress, apoptosis, and autophagy are recognized as the potential mechanisms in Cyclo-induced cardiac toxicity. Through improvements of the antioxidant status (GSH, GPX-1) and histomorphology with downregulations of the oxidative stress marker (MDA) and apoptotic markers (Bax, Bcl2, and caspase-3), TEL and the nanoformulated extract of Sp obtained by ionic gelation are able to mitigate the harmful effects of Cyclo in the cardiac tissues of rats. Additionally, TEL and nano SP show high affinities toward increasing the autophagy signaling pathway via activations of beclin-1 and LC3II while downregulating p62 expression. Hence, the current investigation provides novel evidence for the cardioprotective efficacies of TEL and nano Sp, with greater effects observed in the latter group. With further research to explore the new mechanistic action(s) and careful clinical evaluations, nano Sp is expected to pave the path for a safer and more effective method of managing Cyclo-based therapy while improving patient outcomes.

## Data Availability

The original contributions presented in the study are included in the article/Supplementary material; further inquiries can be directed to the corresponding authors.
